# Inhibition of lung tumor growth by targeting EGFR/VEGFR-Akt/NF-κB pathways with novel theanine derivatives

**DOI:** 10.18632/oncotarget.2336

**Published:** 2014-08-10

**Authors:** Guoying Zhang, Xinshan Ye, Dexin Ji, Huarong Zhang, Fujia Sun, Chunqing Shang, Ying Zhang, Erxi Wu, Fengfei Wang, Fei Wu, Huihui Tian, Xin Liu, Linlin Chen, Kun Liu, Yishan Wang, Hanchen Liu, Wenhua Zhang, Yukun Guan, Qinwen Wang, Xiaohang Zhao, Xiaochun Wan

**Affiliations:** ^1^ Laboratory of Molecular Pharmacology, School of Pharmacy, Yantai University, Qing Quan Lu, Yantai, Shandong Province, China; ^2^ State Key Laboratory of Natural and Biomimetic Drugs, Peking University, Beijing, P R China; ^3^ Department of Pharmaceutical Sciences, North Dakota State University, Fargo, ND, USA; ^4^ The Center of Non-Traumatic Treatment and Diagnosis of Tumor, Binzhou Medical College affiliated The PLA 107 Hospital, Southern Zhichu Road, Yantai, Shandong Province, China; ^5^ Department of Radiotherapy,307 Hospital of PLA,Academy of Military Medical Science,Beijing,P. R. China; ^6^ National Laboratory of Molecular Oncology, Cancer Institute & Hospital, Chinese Academy of Medical Sciences, 17 Panjiayuan, Chaoyangqu, Beijing, P. R. China; ^7^ Key Laboratory of Tea Biochemistry & Biotechnology, Ministry of Agriculture, Anhui Agricultural University, Hefei, Anhui Province, China

**Keywords:** Theanine derivatives, lung cancer, growth and migration, xenograft mouse models, inhibition, EGFR/VEGFR-Akt-NF-κB pathways

## Abstract

The molecularly targeted agents, including anti-VEGF or anti-EGFR monoclonal antibody and some inhibitors of EGFR tyrosine kinase, are effective in the treatment of non-small-cell lung cancer (NSCLC) to a certain extent, but the benefit for a proportion of patients is still limited. Hence, it is necessary and urgent to develop more selective and effective molecular targeted agents against lung cancer. Here, we have synthesized novel theanine derivatives, methyl coumarin-3-carboxylyl L-theanine (TMC), ethyl coumarin-3-carboxylyl L-theanine (TEC), ethyl 6-fluorocoumarin- 3-carboxylyl L-theanine (TFC), and ethyl 6-nitrocoumarin-3-carboxylyl L-theanine (TNC), which are fluorescent small molecules, based on their parental compound theanine and studied their anticancer activities *in vitro*, *ex vivo* and *in vivo* models of human and mouse cancers. Our results show that the four theanine derivatives significantly inhibit the lung cancer cell migration and the growth of lung cancer and leukemia cell lines. TFC and TNC display enhanced effects with anticancer drugs cytarabine, vincristine, and methotrexate on inhibition of lung cancer cell growth and no toxicity to the normal human embryonic lung fibroblast and peripheral blood lymphocytes. TFC and TNC exhibit strong suppression of the highly metastatic Lewis lung cancer (LLC) and A549 tumor growth in tumor-bearing mice without toxicity to mice. TFC and TNC can effectively suppress the growth of lung cancer cells in vitro, ex vivo and in vivo by targeting EGFR/VEGFR-Akt/NF-κB pathways. Our study has suggested that TFC and TNC may have the therapeutic and/or adjuvant therapeutic applications in the treatment of lung cancers and other cancer.

## INTRODUCTION

Lung adenocarcinoma is the leading cause of cancer-related deaths in the world, and non–small cell lung cancer (NSCLC) accounts for nearly 80% of the cases [1-4]. Despite recent advances in diagnosis and treatment, the overall 5-year survival rate of lung cancer patients is only about 15%. The patients with advanced lung cancer have a median survival of approximately 10 months when treated with standard platinum-based therapy. Several newly developed cytotoxic agents including paclitaxel, vinorelbine, irinotecan, and gemcitabine have shown to offer multiple therapeutic choices for patients with advanced NSCLC; however, each of the new regimens can provide only modest improvements in survival and quality of life compared with traditional cisplatin-based therapies [5]. Recently, molecular targeted agents, including anti-EGFR or anti-VEGF monoclonal antibody, cetuximab or bevacizumab, and small molecule inhibitors of EGFR tyrosine kinase, such as erlotinib and gefitinib, have been investigated in clinical trials and/or were approved for clinical use. These agents are effective in the treatment of advanced NSCLC to a certain extent, but a proportion of patients who could receive a survival benefit is still limited [6-7]. Hence, there is an urgent need for more effective therapies and/or molecular targeted anticancer agents for treating patients with lung cancer.

Theanine (*γ*–glutamylethylamide) is a characteristic amino acid found in tea and has been widely used as a safe food additive without limitation to its dose. Reports from different laboratories including our own showed that theanine possesses anticancer activities against some cancers [8-11]. For instance, our previous study showed that theanine and the sera from theanine-fed rats inhibited the invasion of rat hepatoma cells *in vitro* and *ex vivo* [10] and the hepatoma growth as well as metastasis *in vivo* [11]. To develop more effective and lower toxic anticancer agents, here we have synthesized novel theanine derivatives based on the structure of theanine and investigated the effects of these small molecule fluorescent compounds on cancer cell migration, growth, apoptosis, and tumor growth as well as the related receptors-mediated signaling pathways in highly metastatic lung cancer.

## RESULTS

### The synthesized theanine derivatives inhibited lung cancer cell migration and growth of lung cancer and leukemia cells, and induced lung cancer cell apoptosis as well as suppressed the growth of lung cancer stem cells

**Figure 1:**
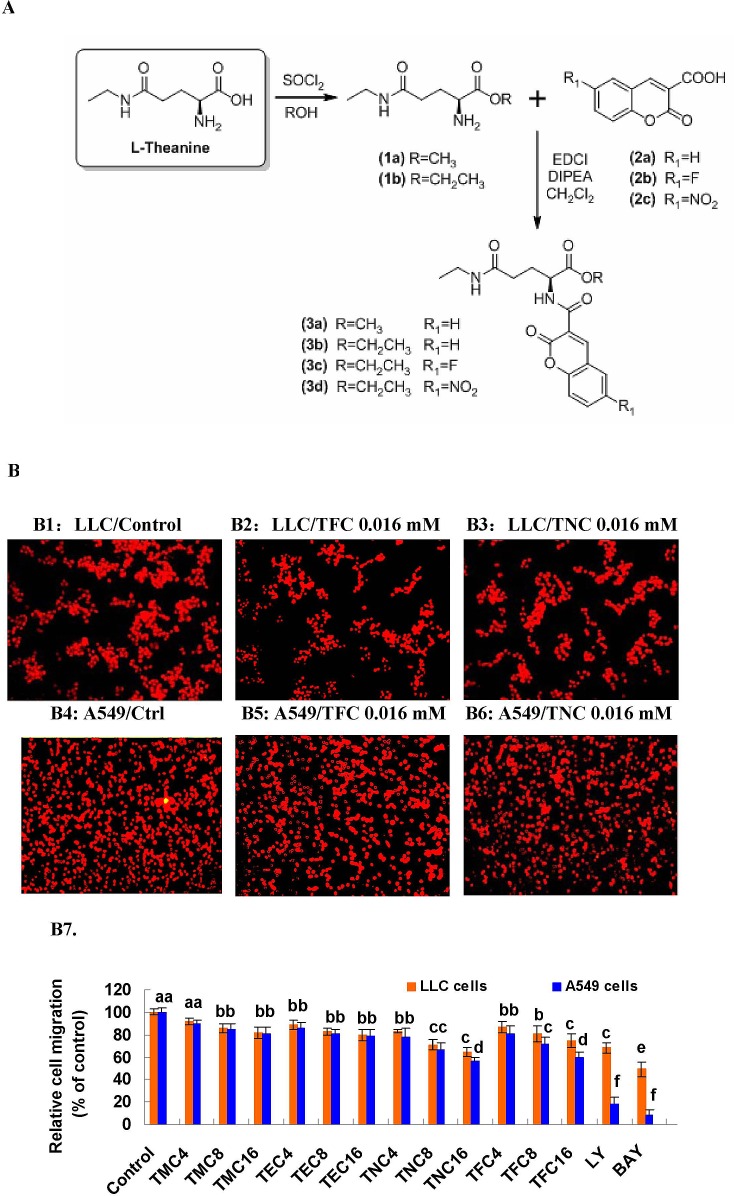
Scheme of synthesis of the four theanine derivatives (3a/TMC, 3b/TEC, 3c/TFC and 3d/TNC) and their inhibitory effects on the migration of lung cancer cell lines **(A)** Scheme of synthesis of the four theanine derivatives. The 3a/TMC, 3b/TEC, 3c/TFC and 3d/TNC were prepared as outlined in the scheme. *L*-Theanine treated with SOCl_2_ in ethanol to generate its methyl ester (1a) or ethyl ester (1b), respectively. The amino group of ester 1a or 1b was acylated with coumarin-3-carboxylic acid (2a), 6-fluorocoumarin-3- carboxylic acid (2b) or 6-nitrocoumarin-3-carboxylic acid (2c) under classical peptide coupling condition to afford the target derivatives 3a−3d. The 3a−3d were purified by column chromatography and the structure was characterized by ^1^H NMR, ^13^C NMR and MS. **(B)** Inhibitory effects of 3a (TMC), 3b (TEC), 3c (TNC) and 3d (TFC) on migration in lung cancer cell lines. The effects of 3a-3d on the migration of LLC and A549 cells were examined by the migration assay as described in the section of “Materials and methods”. The photos (200X) show the propidium iodide-stained LLC (B1, B2, B3) and A549 (B4, B5, B6) cells that migrated through fibronectin-coated transwell chamber. (B7) The quantitative analysis of inhibition of LLC and A549 cell migration by 3a-3d. The cells were treated for 6 h with the indicated concentrations of 3a-3d (0.004–0.016 mM). The control received vehicle. The data are presented as the mean ± SD (Bar) (n=6). The figure is the representative of 3 similar experiments performed. Values with different letters (a–f) differ significantly (*P* < 0.05).

In this study, we synthesized four novel theanine derivatives which are small molecule fluorescent compounds, methyl coumarin-3-carboxylyl L-theanine (MCCT, short for TMC/3a), ethyl coumarin-3-carboxylyl L-theanine (ECCT, short for TEC/3b), ethyl 6-fluorocoumarin-3-carboxylyl L-theanine (EFCT, short for TFC/3c), and ethyl 6-nitrocoumarin-3-carboxylyl L-theanine (ENCT, short for TNC/3d), based on their parental compound theanine targeting the migration and growth of cancer cells. The scheme of theanine derivatives (3a/TMC, 3b/TEC, 3c/TFC, 3d/TNC) synthesis and chemical structures are shown in Fig. 1A. The numbers of application for national patents in China and for an international patent are 201210363367.0, 201210363378.9, 201210515826.2, 201210515827.7, and PCT/CN2013/084146, respectively. In previous studies, including our own, theanine displayed some anticancer activities [8-11]. Because the high water solubility of theanine and the structure of coumarin-3-carboxylic acid could limit the antitumor activity *in vitro* and *in vivo*, we synthesized the theanine derivatives, 3a/TMC, 3b/TEC, 3c/TFC, and 3d/TNC by esterification of the carboxyl group of theanine with ethanol and acylation of the amino group of theanine with 6-substituted coumarin-3-carboxylic acid. We hypothesized and confirmed that 3a/TMC, 3b/TEC, 3c/TFC, and 3d/TNC would greatly enhance the anticancer activity *in vitro* and *in vivo*.

We first tested the effects of 3a/TMC, 3b/TEC, 3c/TFC, and 3d/TNC on tumor cell migration. Our results showed that TMC, TEC, TFC and TNC (0.004 to 0.016 mM) significantly suppressed the migration of highly metastatic Lewis lung cancer (LLC) and A549 cells in a dose-dependent manner (Fig. 1B). The ratios of lung cancer cell migration were reduced by 18%, 20%, 25%, and 35% in LLC cells, and by 19%, 21%, 40%, and 44% in A549 cells, respectively in response to the treatment of 0.016 mM of TMC, TEC, TFC and TNC, although 24 h treatment with TMC, TEC, TFC and TNC at the same concentration did not significantly affect the growth of both LLC and A549 cells (data not shown).

**Figure 2:**
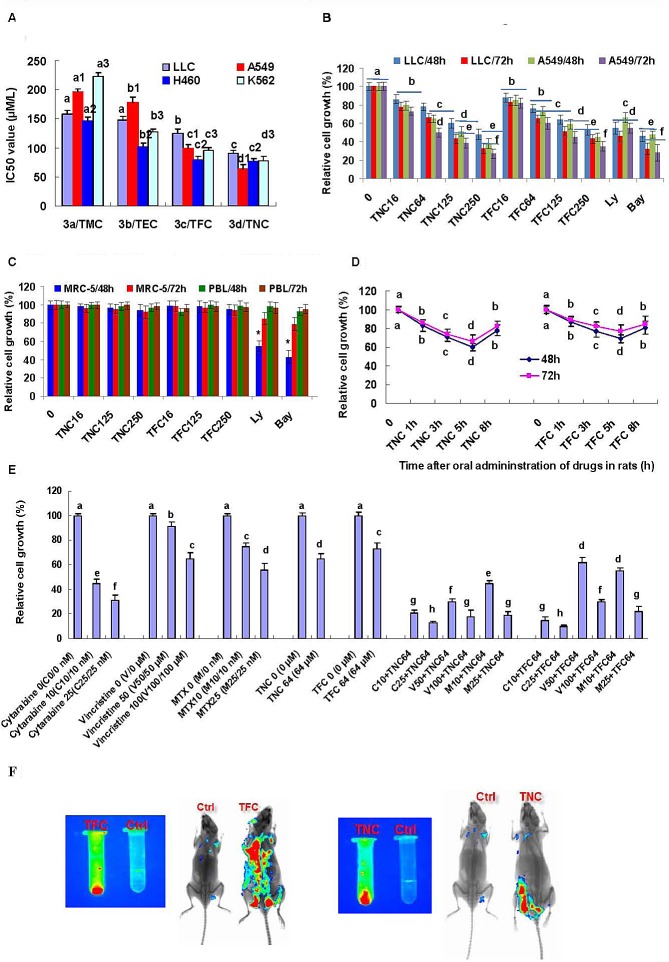
Effects of theanine derivatives on the growth of cancer cell lines and normal cells **(A)** Theanine derivatives TMC, TEC, TFC and TNC showed growth inhibition of lung cancer LLC, A549 and H460 cell lines as well as leukemia K562 cell line with the indicated IC50 values (72h treatment). **(B)** TFC and TNC significantly inhibited the growth of LLC and A549 cells in dose- and time-dependent manners. **(C)** TFC and TNC showed no significant effect on growth of normal human embryonic lung fibroblast MRC-5 and peripheral blood lymphocytes (PBL). **(D)** TFC and TNC exhibited ex vivo growth inhibition of A549 cells by the sera from TFC- or TNC-treated rats at 1, 3, 5 and 8 h after oral administration of TFC or TNC in rats. **(E)** TFC and TNC (64 μM) enhanced the growth inhibition of A549 cells in combination with anticancer drugs cytarabine (10-25 nM), vincristine (50-100 μM), and methotrexate (10-25 nM) (72h treatment). The cells were treated for 48 h and/or 72 h with the indicated concentrations of TFC or TNC (TFC 16–TNC 250/0.016–0.25 mM), Ly (0.016 mM), and Bay (0.0032 mM). The cells in control group were treated with DMSO vehicle. The rate of relative cell growth was determined by the MTT assay. Ly294002 (Ly) and Bay are the inhibitors of PI3K/Akt and NF-*κ*B, respectively. The data are presented as the mean ± SD (Bar) (n=6). **(F)** TFC and TNC showed the *in vitro* (left; 8 mg/ml) and *in vivo* (right) fluorescent signals in mice (3 h after injection of TFC or TNC at 80 mg/kg body weight, or DMSO vehicle). The figures are the representative of 3 similar experiments performed. Values with different letters (a–h; a, a1, a2, a3 – d, d1, d2, d3) differ significantly (*P* < 0.05).

We next tested the effects of TMC, TEC, TFC and TNC on the growth in cancer cell lines. The result indicated that 48 h and 72 h treatment with TMC, TEC, TFC and TNC significantly suppressed the growth in the lung cancer and leukemia cells. Their IC50 values (72 h treatment) of growth inhibition are 0.158, 0.148, 0.125, and 0.09 mM for LLC cells, 0.196, 0.179, 0.99, and 0.064 mM for A549 cells, 0.147, 0.102, 0.079, and 0.076 mM for H460 cells, and 0.223, 0.127, 0.096, and 0.078 mM for K562 cells, respectively (Fig 2A). Then, we focused on studying the effects of TNC and TFC on the growth of LLC and A549 cells, based on the results of the IC50 values and migration inhibition by the four theanine derivatives. Both of TNC and TFC at the concentrations of 0.016 to 0.25 mM significantly inhibited the growth of lung cancer LLC and A549 cell lines in dose- and time-dependent manners (Fig. 2B). Moreover, TNC and TFC at the same concentrations of 0.016 to 0.25 mM had little effect on the growth of the normal human embryonic lung fibroblast MRC-5 and human peripheral blood lymphocytes (PBL) (Fig. 2C). In contrast, the inhibitors of PI3K/Akt (Ly294002, 0.016 mM) and NF-*κ*B (Bay, 0.0032 mM), used as positive controls, significantly inhibited the growth of not only lung cancer cell lines but also normal MRC-5 cells (Fig. 2B, 2C).

We previously reported that theanine displayed significant inhibition of cancer cell growth at 1 h and the inhibitory peak of cancer cell growth at 1 h after its oral administration in rats [10]. In contrast, our present data show that the synthesized theanine derivatives TNC and TFC exhibits the significant inhibition of cancer cell growth for 8 h and the inhibitory peak of cancer cell growth at 5 h after their oral administration in rats (Fig. 2D). More importantly, TNC and TFC can significantly enhance the suppression of A549 cell growth in combination with anticancer drugs cytarabine, vincristine, and methotrexate (Fig. 1E). In addition, TNC and TFC show the very strong *in vitro* and *in vivo* fluorescence characteristics. As shown in Fig. 2F, TNC and TFC display strong fluorescent signaling *in vitro* and fluorescent distribution of TNC and TFC can be observed in mouse tissues 3 h after their introperitoneal injection.

**Figure 3:**
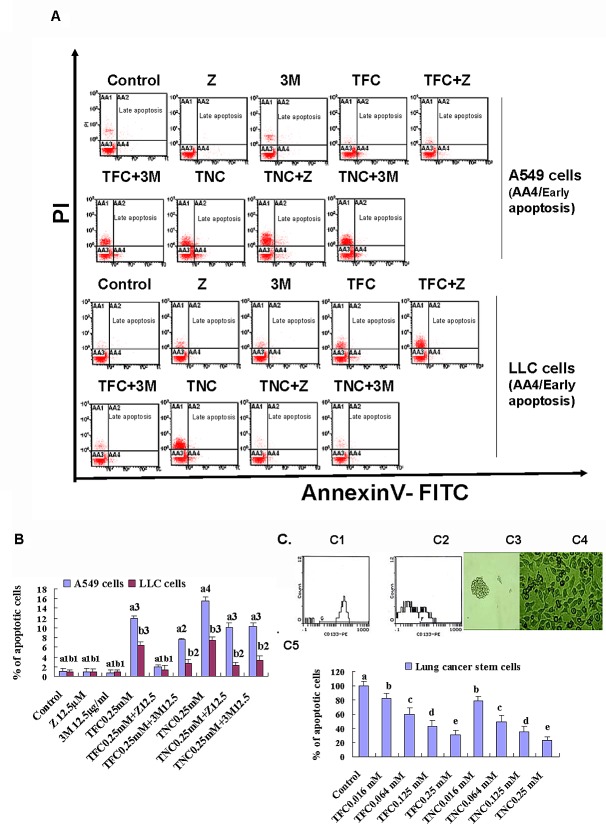
Effects of TFC and TNC on apoptosis in highly-metastatic Lewis lung cancer (LLC) and A549 cells and lung cancer stem cells (CSCs) growth **(A)** FACS analysis showed the apoptosis in LLC and A549 cells after the cells were treated for 24 h with the indicated concentrations of TFC (0.25 mM), TNC (0.25 mM), a caspase inhibitor Z-VAD-FMK (Z, 12.5 μM), an autophagic inhibitor 3-methyladenine (3M, 12.5 μg/ml), TFC+Z, TFC+3M, TNC+Z, and TNC+3M. **(B)** The summary of total percent apoptosis (early apoptosis/AA4 plus late apoptosis/AA3) in the cells treated with TFC, TNC, Z, 3M, TFC+Z, TFC+3M, TNC+Z, and TNC+3M Values with different letters (a1–a4: a1, a2, a3, a4; b1-b3: b1, b2, b3) differ significantly (*P* < 0.05). (C) FACS analysis indicated that the positive CD133 expression was 65.6% and 15.5% in the lung CSCs (C1) and the parental A549 cells (C2), respectively. The photos (200X) exhibited the CSCs spheres (C3) and A549 cells (C4). (C5) MTT assay confirmed that the CSCs growth was significantly inhibited by 4-day treatment with TFC and TNC at concentrations of 0.016 to 0.25 mM. Values with different letters (a–e) differ significantly (*P* < 0.05).

In order to determine the mechanisms of growth inhibition by TNC and TFC in LLC and A549 cells, we analyzed their effects on the cell apoptosis using FACS by Annexin V-FITC/PI double- staining assay. The results indicated that TNC and TFC at 0.25 mM displayed evident induction of apoptosis in LLC and A549 cells after the cells were treated for 24 h. In response to the treatment of TNC and TFC at 0.25 mM, the apoptotic ratios were 7.5% and 6.5% in LLC cells, and 15.6% and 11.9% in A549 cells, respectively (Fig. 3A, 3B). In addition, a caspase inhibitor Z-VAD-FMK (Z, 12.5 μM) reduced most apoptosis in TNC- and TFC-treated LLC and A549 cells (Fig. 3A, 3B) although an autophagic inhibitor 3-methyladenine (3M, 12.5 μg/ml) partially reduced the apoptosis in the TNC- and TFC-treated LLC and A549 cells. The induction of apoptosis in LLC and A549 cells by TNC and TFC could greatly contribute to the growth inhibition in the lung cancer cells (Fig. 2A, 2B). Moreover, TNC and TFC at the concentrations of 0.016 to 0.25 mM significantly inhibited the growth of lung cancer stem cells after 4-day treatment (Fig.3C).

### TFC and TNC down-regulated the protein levels of Bcl-2 and cyclin D1 and up-regulated the protein levels of Bax, cytosolic cytochrome c, caspase-3, PARP-1, p53, and p21 in LLC cells

**Figure 4:**
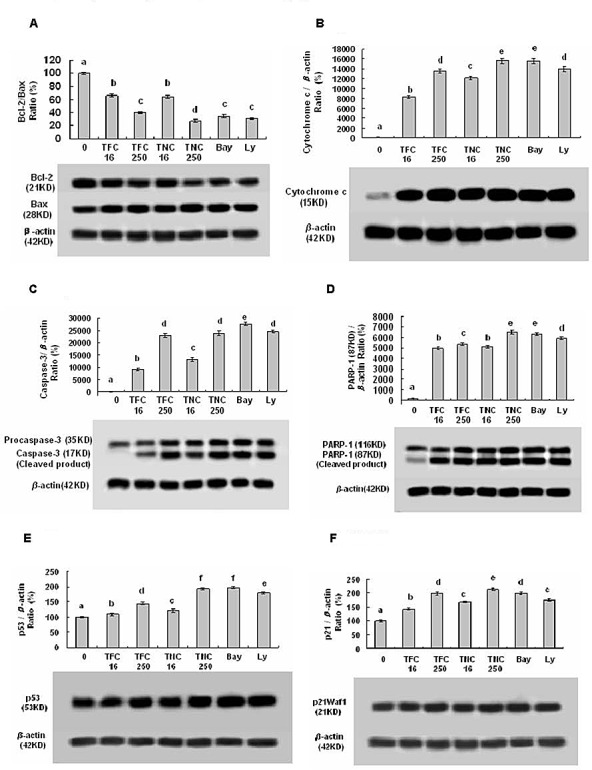
Effects of TFC and TNC on protein expressions of Bcl-2/Bax (A), cytosolic cytochrome c (Cyto c) (B), caspase-3/pro-caspase-3 (C), PARP-1 (D), p53 (E), and p21 (F) in highly-metastatic LLC cells The cells were treated for 48 h with the indicated concentrations of TFC and TNC (TFC16/TNC16– TFC250/TNC250: 0.016–0.25 mM), Ly (0.016 mM) and Bay (0.0032 mM). The protein expressions were analyzed by Western Blotting. The optical density (OD) of the band is normalized with respective β-actin and is expressed as relative optical density (OD). The OD value of the band shown as mean ± SD is relative to that of the control (DMSO vehicle) designated as 100%. Bay and Ly are the inhibitors of NF-*κ*B and PI3K/Akt, respectively. For one experiment, 3 assays were carried out and only one set of gels is shown. Values with different letters (a–f) differ significantly (*P* < 0.05).

**Figure 5:**
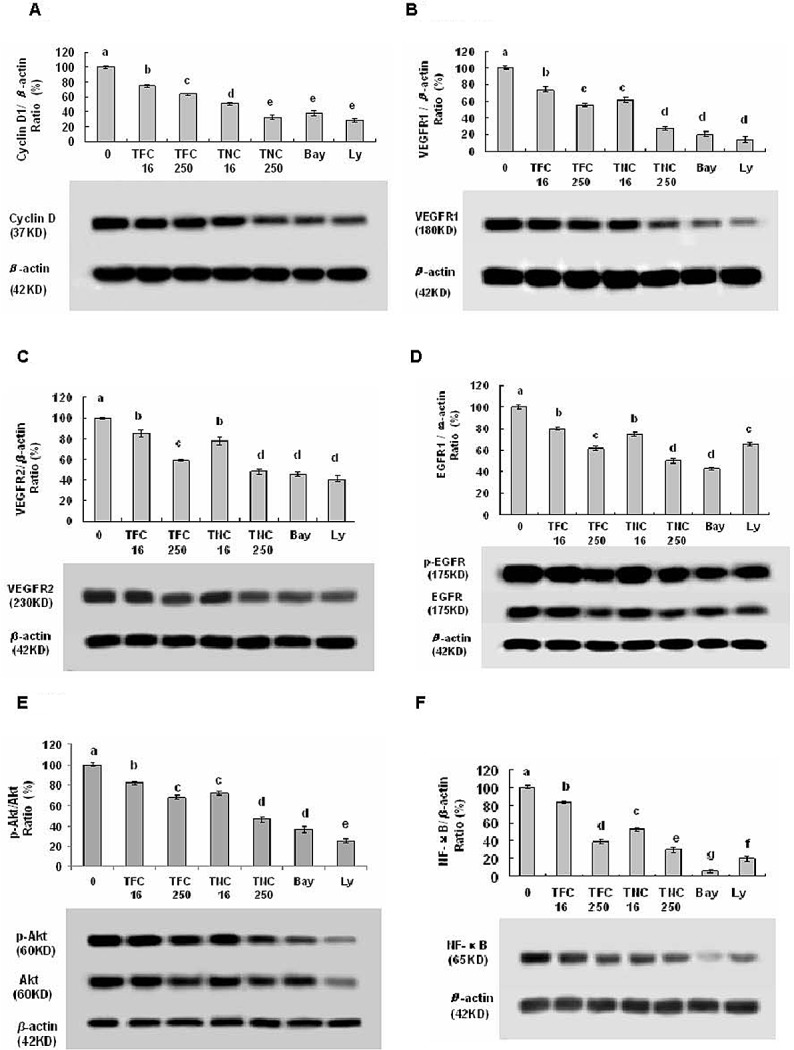
Effects of TFC and TNC on protein expressions and/or phosphorylation of cyclin D1 (A), VEGFR1 (B), VEGFR2 (C), pEGFR/EGFR (D), pAkt/Akt (E), and NF-κB (F) in highly metastatic LLC cells The cells were treated for 48 h with the indicated concentrations of TFC and TNC (TFC16/TNC16–TFC250/TNC250: 0.016–0.25 mM), Ly (0.016 mM) and Bay (0.0032 mM). The protein expressions were analyzed by Western Blotting. The optical density (OD) of the band is normalized with respective β-actin and is expressed as relative optical density (OD). The OD value of the band shown as mean ± SD is relative to that of the control (DMSO vehicle) designated as 100%. Bay and Ly are the inhibitors of NF-*κ*B and PI3K/Akt, respectively. For one experiment, 3 assays were carried out and only one set of gels is shown. Values with different letters (a–g) differ significantly (*P* < 0.05).

To understand the molecular mechanisms by which TFC and TNC inhibit cell growth and induce apoptosis in lung cancer cells, we studied the effects of TFC and TNC on the expressions of proteins related to cell growth and apoptosis as well as cell cycle arrest in LLC cells. Our results showed that TFC and TNC markedly reduced the Bcl-2 protein levels in LLC cells at the concentrations of 0.016 to 0.25 mM (Fig. 4A). Furthermore, TFC and TNC remarkably up-regulated the expression levels of Bax (Fig. 4A), cytosolic cytochrome c (Fig. 4B), caspase-3 (Fig. 4C), the cleavage of poly(ADP-ribose) polymerase-1 (PARP-1) (Fig. 4D), p53 (Fig. 4E), and p21 WAF1 (Fig. 4F) in LLC cells. In addition, TFC and TNC greatly reduced the protein expression levels of cyclin D1 (Fig. 5A) in LLC cells. TNC showed much stronger activity than TFC on the regulation of the protein expressions of Bcl-2/Bax ratio, PARP-1, p53, and cyclin D1 in LLC cells.

### TFC and TNC down-regulated the phosphorylation and/or protein expressions of VEGFR1, VEGFR2, EGFR, Akt, and NF-κB in LLC cells

In order to clarify the receptors regulated in TFC- and TNC-treated LLC cells, we studied the effects of TFC and TNC on the related receptor phosphorylation and expressions in LLC cells. Our results show that TFC and TNC at the concentrations of 0.016 to 0.25 mM remarkably reduced the expressions of VEGFR1 (Fig. 5B) and VEGFR2 (Fig. 5C), and the phosphorylation and expressions of EGFR (Fig. 5D) and Akt (Fig. 5E) in LLC cells. Moreover, TFC and TNC greatly reduced NF-κB protein expression (Fig. 5F) in LLC cells. TNC showed much stronger activity than TFC on the regulation of the protein expressions of VEGFR1, pAkt and Akt in LLC cells.

### TFC and TNC suppressed TNFα- and EGF+HGF-enhanced growth and/or NF-κB signaling in A549 cells

**Figure 6:**
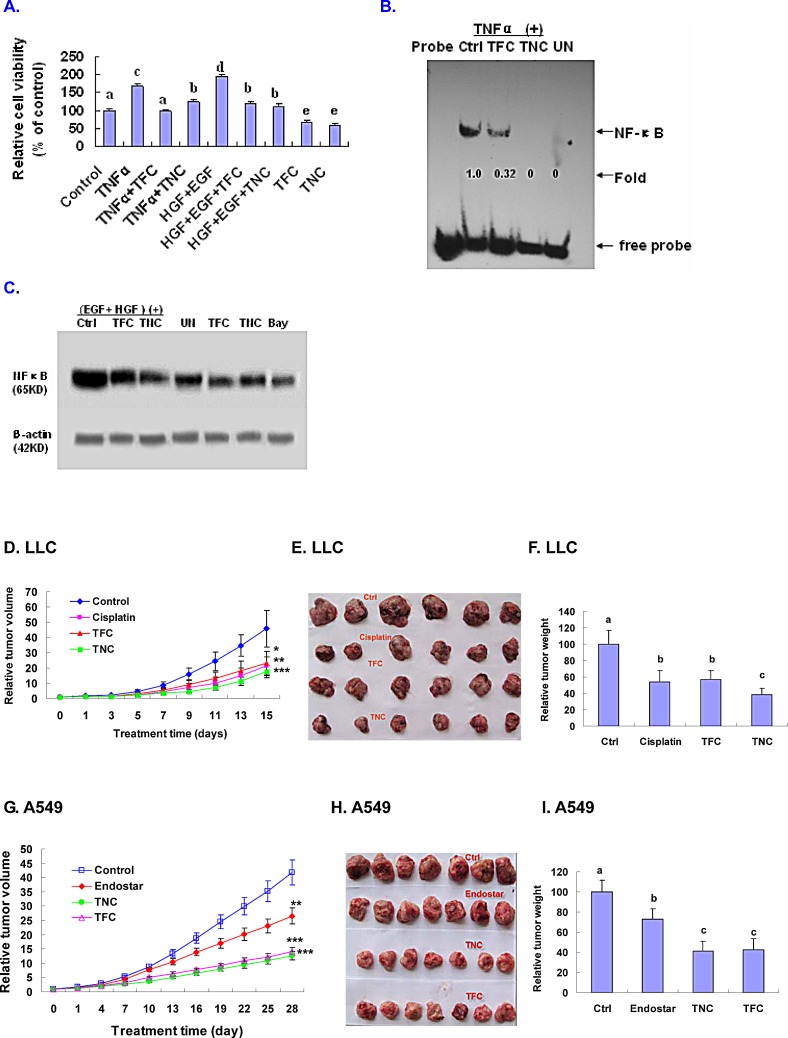
Suppression of TNFα- and EGF+HGF-enhanced growth and/or NF-κB signaling in A549 cells and ***in vivo*** inhibition of LLC and A549 xenografts tumor growth in tumor-bearing mice by TFC and TNC **(A)** Tryban blue exclusion test confirmed that TFC and TNC at 0.25 mM significantly suppressed TNFα- (20 ng/ml) and EGF+HGF- (100 ng/ml) enhanced growth in A549 cells after 24 h treatment; Values with different letters (a–c) differ significantly (*P* < 0.05). **(B)** A549 cells were treated for 30 minutes with TFC and TNC at 0.25 mM. After 6 h of TNF-α treatment, the treated cell nuclear extracts were used to determine the NF-κB DNA-binding activity by EMSA. UN, untreated cells; **(C)** After A549 cells were pretreated for 30 minutes with TFC and TNC at 0.25 mM, the cells were treated with EGF (100 ng/ml) +HGF (100 ng/ml) or without EGF+HGF treatment for 24 h. The protein expression of NF-κB in A549 cells were analyzed by Western blotting. UN, untreated cells; Bay, the inhibitor of NF-κB (0.005 mM); **(D-I)** *In vivo* inhibition of LLC and A549 xenografts tumor growth. LLC cells (0.6 × 10^6^) and A549 cells (3 × 10^6^) were xenotransplanted into the rear right flank of each mouse, respectively. After 10-15 days, the tumor models were established, respectively. The tumor-bearing mice were randomly divided into four groups: (1) control group (vehicle); (2) cisplatin group for LLC tumor treatment (1.5 mg/kg/day; 6 days each week); endostar group for A549 tumor treatment (7 mg/kg/day; 6 days each week); (3) TFC group (TFC 80 mg/kg/day); (4) TNC group (TNC 80 mg/kg/day). The mice were treated with vehicle, cisplatin (for LLC), endosatr (for A549), TFC and TNC by introperitoneal injection once every day. The therapy was performed for 15 days (in the case of LLC tumors) or 28 days (in the case of A549 tumors). **(D-F)** Suppression of highly metastatic LLC tumor growth by TFC and TNC. **(G-I)** Inhibition of A549 human lung tumor growth by TFC and TNC. Tumor volumes were measured once every 2 days (LLC) or 3 days (A549) and relative tumor volumes [31] at each time point were calculated as tumor volume at that time divided by the tumor volume at the beginning of treatment (0 day). The relative tumor weight is relative to that of the control (vehicle) designated as 100. Tumor volumes and weights were compared among groups using the ANOVA and Bonferroni post-test. Values are shown as mean ± SD for each group (n=6 and n=7 for LLC and A549, respectively). Values with different letters (a–c) differ significantly (*P* < 0.05). All statistical tests were two-sided.

In order to approve that down-regulation of NF-κB activity is essential for TFC and TNC's anticancer effect, we tested effects of TFC and TNC on the growth and NF-κB protein expression and DNA-NF-κB binding activity in A549 cells stimulated with TNFα and EGF+HGF. We confirmed that TFC and TNC at 0.25 mM significantly suppressed TNFα- (20 ng/ml) and EGF+HGF- (100 μg/ml) enhanced growth in A549 cells after 24 h treatment (Fig. 6A). Moreover, TFC and TNC at 0.25 mM remarkably reduced TNFα-enhanced DNA-NF-κB binding activity in A549 cells (Fig.6B). In addition, TFC and TNC at 0.25 mM significantly decreased the EGF+HGF-enhanced NF-κB protein expression in A549 cells (Fig.6C).

### TFC and TNC suppressed tumor growth in tumor-bearing mice

The animal experimental results showed that TFC and TNC induced time-dependent inhibition of tumor growth in the highly metastatic LLC and A549 tumor-bearing mice. The relative inhibitory rates of tumor volumes (Fig. 6D, 6E) and weights (Fig. 6F) in LLC decreased by 50.2% and 43.0% (for TFC treatment), and 60.5% and 61.4% (for TNC treatment), respectively after treatment with TFC and TNC for 15 days in LLC tumor-bearing mice. In the case of A549 tumor-bearing mice, the relative inhibitory rates of tumor volumes (Fig. 6G, 6H) and weights (Fig. 6I) were reduced by 65.0% and 57.3% (for TFC treatment), and 68.2% and 58.8% (for TNC treatment), respectively after treatment with TFC and TNC for 28 days. As the positive controls, the anticancer drugs cisplatin and endostar also displayed significant inhibition of the tumor growth of LLC (Fig. 6D, 6E, 6F) and A549 (Fig. 6G, 6H, 6I), respectively in the tumor-bearing mice. In addition, TFC and TNC showed no evidence of toxicity to the mice as measured by body weights and the lack of pathological changes in the mice at autopsy. The LD50 for mice treated with TFC and TNC is 3000 ±253 mg/kg and 3500 mg/kg ± 226 (body weight), respectively.

## DISCUSSION

In the present study, we have synthesized the four theanine derivatives TMC, TEC, TFC and TNC, and confirmed that both TFC and TNC have the very strong *in vitro* and *in vivo* fluorescence characteristics (Fig. 2F). We have demonstrated that TMC, TEC, TFC and TNC can significantly inhibit the migration (Fig. 1B) of LLC and A549 cells, and the growth (Fig. 2) of LLC, A549, H460 and K562 cells. We have also indicated that TFC and TNC can repress the *ex vivo* growth of A549 cells (Fig. 2D) and induce apoptosis in LLC and A549 cell lines as well as inhibit lung CSCs in a dose-dependent manner (Fig. 3). In addition, we have shown that TFC and TNC can significantly enhance the effects in combination with anticancer drugs cytarabine, vincristine, and methotrexate on suppression of A549 cell growth (Fig. 2E). In order to understand the molecular mechanisms of action, we further examined the protein expressions related to the migration, growth, apoptosis in the highly metastatic LLC cells.

One of the main regulatory steps of apoptotic cell death is controlled by the ratio of antiapoptotic and proapoptotic members of the Bcl-2 family of proteins, which determines the susceptibility to apoptosis. Bax, a proapoptotic factor of the Bcl-2 family, is found in monomeric form in the cytosol or is loosely attached to the membranes under normal conditions. Following a cell death stimulus, cytosolic and monomeric Bax translocates to the mitochondria where it becomes an integral membrane protein and cross-linkable as a homodimer allowing for the release of factors, such as cytochrome c, from the mitochondria to propagate the apoptotic pathway [12-13]. Here, we observed that TFC and TNC significantly down-regulated the antiapoptotic Bcl-2 level and up-regulated the proapoptotic Bax level, leading to reduction of Bcl-2/Bax ratio (Fig. 4A). Furthermore, TFC and TNC significantly enhanced the release of cytocrome c from the mitochondria (Fig. 4B) and subsequently increased the caspase-3 activity (Fig. 4C). Caspase-3 is synthesized as a 35-kDa inactive precursor (procaspase-3), which is proteolytically cleaved to produce a mature enzyme composed of 17-kDa fragments. As shown in Figure 4C and 4D, the protein levels of caspase-3 in cleaved form and cleaved PARP-1, the substrate of caspase-3 were enhanced with the increase in the concentrations of TFC and TNC. In addition, we observed the up-regulation of tumor suppressor p53 (Fig. 4E) and endogenous cyclin-dependent kinase inhibitor p21 WAF1/CIP1 (Fig. 4F) in LLC cells treated with TFC and TNC. Moreover, our results also indicated that TFC and TNC significantly down-regulated the cyclin D1 protein expression in LLC cells (Fig. 5A). Taken together, these results showed that TFC and TNC significantly suppressed the growth of LLC cells and remarkably induced the apoptosis by reducing Bcl-2/Bax ratio, activating the mitochondrial and caspase-3 pathways, and enhancing p53 and p21 WAF1 protein levels as well as decreasing the protein level of cyclin D1, the cell cycle regulator in LLC cells.

The activated receptors such as VEGFR1, VEGFR2, and EGFR play very important roles in the migration and proliferation of cancer cells including lung cancer cells [14-20]. The down-regulation of the receptor phosphorylation and/or protein expressions in LLC cells (Fig. 5B, 5C, 5D) by TFC and TNC could greatly contribute to the suppression of their downstream targets and the receptor-mediated signaling pathways, and thus would lead to the inhibition of the migration and growth in the lung cancer cells. In order to further confirm the inhibitory effects of TFC and TNC on the signaling pathways related to the migration and growth of the lung cancer cells, we investigated the effects of TFC and TNC on the suppression of the phosphorylation and/or expressions of Akt and NF-κB in LLC cells. Our results indicated that TFC and TNC significantly reduced the phosphorylation and expression of Akt (Fig. 5E) and the NF-κB (p65) expression (Fig. 5F) in LLC cells. In addition, the inhibitors of PI3K/Akt (Ly) and NF-κB (Bay) displayed the significant suppression of the phosphorylation and protein expression of Akt and the NF-κB (p65) expression in LLC cells,respectively (Fig. 5E, 5F). Akt is a cytosolic signal transduction protein kinase that plays an important role in cell survival pathways [21]. Induction of Akt activity is primarily dependent on the PI3K pathway. Akt can be activated by many forms of cellular stress, such as those observed under treatment with anticancer agents [21]. Once activation, Akt controls cellular functions such as cell cycle, apoptosis, gene transcription, and protein synthesis through the phosphorylation of downstream substrates such as NF-*κ*B [21]. NF-*κ*B is a nuclear transcription regulator with a specific motif for Bcl-2 transcription [22-24]. Activation of p-Akt and the NF-*κ*B/Bcl-2 pathway leads to inhibition of chemotherapy-induced apoptosis, which results in treatment resistance [22]. Our previous results confirmed that suppression of Akt and the NF-*κ*B/Bcl-2 pathway by anticancer agents inhibited the migration and growth as well as induced the apoptosis in human breast and lung cancer cells [19-20; 23; 30]. Here, we demonstrated that TFC and TNC significantly suppressed TNFα- and EGF+HGF-enhanced growth and/or NF-κB signaling in A549 cells. Our present study indicated that TFC and TNC significantly inhibited the migration and growth, and induced apoptosis in the lung cancer cells as well as suppressed the PI3K/Akt and NF-κB pathway by reducing the phosphorylation and expression of Akt and the NF-κB expression and DNA-binding activity, and activating the apoptotic pathway of Bcl-2/Bax-mitochondrial- caspase-3 in the lung cancer cells.

EGFR, VEGFR, Akt, and NF-kB have become the important targets against a variety of cancer including lung cancer cells [14-20; 23; 30]. We have demonstrated that TFC and TNC significantly inhibited their phosphorylation and/or expressions and their downstream targets. Suppression of these receptors-activated Akt-NF-κB signaling pathways by TFC and TNC could greatly contribute to the inhibition of the migration and growth of lung cancer LLC and A549 cells as well as the LLC and A549 tumor growth in tumor-bearing mice as we observed in the present study.

In summary, we have shown that the synthesized novel small molecule fluorescent compounds TMC, TEC, TFC and TNC have very strong activities against cancer. Our results have demonstrated that TMC, TEC, TFC and TNC not only inhibit the growth of human H460 and K562 cancer cell lines, but also significantly suppresses the LLC and A549 cell migration and growth. TFC and TNC can induce the LLC and A549 cell apoptosis and inhibit lung CSCs growth. We have also shown that TFC and TNC can suppress the VEGFR1, VEGFR2, and EGFR receptors-mediated signaling pathways of Akt-NF-κB in LLC cells. TFC and TNC do not inhibit the growth of the normal human embryonic lung fibroblast MRC-5 and human peripheral blood lymphocytes. TFC and TNC also do not display any toxicity to the mice, although both of them have the strong suppression of the lung tumor growth in the LLC and A549 tumor-bearing mice. In addition, TFC and TNC significantly enhance the inhibition of cancer cell growth when combined with conventional anticancer drugs. All these findings suggest that TFC and TNC as novel anticancer compounds may have the therapeutic and/or adjuvant therapeutic applications in the treatment of lung cancer and other cancers.

## MATERIALS AND METHODS

### Chemicals and antibodies

The primary antibodies to mouse Bcl-2, Bax, caspase-3, PARP-1, cytochrome c, cyclic D1, p21WAF1, p53, NF-κB (p-65), Akt, phospho-Akt (Ser473), VEGFR2, EGFR and phospho-EGFR (Tyr1068), were purchased from Cell Signaling Technology Inc. (Beverley, MA, USA). The primary antibodies to mouse VEGFR1 and β-actin were purchased from Santa Cruz Technology Inc. (Dallas, Texas, USA). Fibronectin and Boyden chambers were obtained from BD Inc. (BD Biosciences, San Jose, CA, USA) and Corning Inc. (Corning, NY, USA), respectively. Cisplatin, endostar, cytarabine, vincristine, and methotrexate were purchased from Yuhuangding Hospitai, Yantai, China. Annexin V-FITC/PI Apoptosis Detection Kit and Z-VAD-FMK were purchased from Beyotime, Haimen, China;DMEM, RPMI 1640 medium, penicillin, streptomycin, fetal bovine serum (FBS), trypsin/EDTA, propidium iodide, 3-[4,5-Dimethylthiazol-2-yl]-2,5-diphenyltetrazolium bromide (MTT), DMSO, Ly294002 (Ly, Bay 11-7082 (Bay), theanine, 3-methyladenine, and all other chemicals including materials for the synthesis of TMC, TEC, TFC and TNC were acquired from Sigma Chemical Co. (St. Louis, MO, USA).

### Preparation of theanine derivatives 3a/TMC, 3b/TEC, 3c/TFC and 3d/TNC

The scheme of synthesis of the theanine derivatives 3a/TMC, 3b/TEC, 3c/TFC and 3d/TNC was shown in Fig. 1A. The detailed procedure for the synthesis of 3a/TMC, 3b/TEC, 3c/TFC and 3d/TNC was shown as follows.

#### General

All the material we used were purchased from commercial suppliers and used without further purification, unless otherwise noted. Solvents were distilled prior to use and flash chromatography was performed using silica gel (200-300 mesh). 6-Fluorocoumarin-3-carboxylic acid (2b) was synthesized according to the literature method [25]. Reactions were routinely monitored by thin-layer chromatography on 0.25 mm silica gel plates (60 GF-254) and visualized with ultraviolet lamp (254 nm) or Ninhydrin Spray (0.5% in butanol). Melting points were determined on an electrothermal melting point apparatus, and the thermometer was uncorrected. ^1^H, ^13c^ NMR spectra were determined on a Bruker Avance III spectrometer using TMS as an internal standard in DMSO-*d*_6_ or CDCl_3_ solutions, operating at a frequency of 500 MHz for proton and 125 MHz for carbon. Peak positions are given in parts per million (δ) from tetramethylsilane, and coupling constant value (*J*) are given in Hz. ESI-MS were determined on an API 4000 spectrometer. All chemicals were purchased from Sigma Chemicals Ltd.

### General procedure for the synthesis of L-theanine esters 1a and 1b

To a solution of *L*-Theanine (87mg, 0.5 mmol) in absolute methanol or ethanol (1 mL), SOCl_2_ (0.055 ml, 0.76 mmol) was added slowly. The reaction mixture was stirred at room temperature for about 2 hours, the end of the reaction was observed by TLC. The resulting mixture was concentrated under reduced pressure to give crude product of *(S)-*methyl 2-amino-5-(ethylamino)-5-oxopentanoate (1a) or *(S)-*ethyl 2-amino-5- (ethylamino)-5-oxopentanoate (1b), respectively. The crude products were used in the next step without further purification.

### Preparation of 6-nitro-2-oxo-2H-chromene-3-carboxylic acid (2c)

To a solution of 2-oxo-2H-chromene-3-carboxylic acid (104 mg, 0.5 mmol) in concentrated sulfuric acid (0.9 ml), cooling under -10 °C, 65% nitric acid (0.3 ml) was added slowly, and the mixture was stirred at room temperature for 1 hour. The solution was then poured into cold water. The resulting light yellow solid was collected by filtration, washed with water and dried under vacuum for 2 hours. 6-Nitro-2-oxo-2H-chromene-3- carboxylic acid (2c) was obtained as light yellow solid, 122 mg, yield 94.8%, m.p. = 234−235 °C (literature value 235 °C) [26].

### General Procedure for the Synthesis of amide derivatives 3a-3d

The functionalized 2-oxo-2H-chromene-3-carboxylic acid 2a−2c (1 mmol) was added to a solution of the crude *L*-theanine ester obtained before (ca. 0.5 mmol) in dry CH_2_Cl_2_ (20 mL). DIPEA (0.61 mmol) and EDCI (0.2 mol) were added to the mixture subsequently. The reaction mixture was stirred for about 1 hour at room temperature, the end of the reaction was observed by TLC. Water (20 mL) was added. After a few minutes stirring, the resulting mixture was extracted with EtOAc (3×10 mL). The organic phase was washed with brine (10 mL), dried over anhydrous MgSO_4_, filtered and evaporated. The residue was purified by flash chromatography (16.7% acetone/n-hexane as eluant) on silica gel. The products were recrystallized from a mixture of acetone and n-hexane, affording the amide derivatives 3a−3d.

*(S)-Methyl 5-(ethylamino)-5-oxo-2-(2-oxo-2H-chromene-3-carboxamido) pentanoate* (3a). White solid, m.p. = 154−156 °C, Yield: 76.6%. ^1^H-NMR (CDCl_3_): 1.15 (t, 3H, *J* = 7.2 Hz, CH_3_CH_2_NH), 2.10–2.17 (m, 1H, COCH_2_CH_2_CHCO), 2.28–2.31 (m, 2H, COCH_2_CH_2_CHCO), 2.34–2.41 (m, 1H, COCH_2_CH_2_CHCO), 3.27–3.33 (m, 2H, CH_3_CH_2_NH), 3.78 (s, 3H, OCH_3_), 4.78–4.83 (m, 1H, COCH_2_CH_2_CHCO), 6.07 (br s, 1H, NH), 7.38–7.43 (m, 2H, chromene H6, H8), 7.67–7.71 (m, 2H, chromene H5, H7), 8.88 (s, 1H, chromene H4), 9.35 (br d, 1H, *J* = 7.6 Hz, NH). ^13^C NMR: 14.8 (CH_3_CH_2_NH), 28.7 (COCH_2_CH_2_CHCO), 32.6 (COCH_2_), 34.5 (CH_2_NH), 52.3 (OCH_3_), 52.6 (CHCO), 116.7 (chromene C8), 117.9 (chromene C3), 118.5 (chromene C4α), 125.4 (chromene C4), 129.9 (chromene C6), 134.4 (chromene C5), 148.8 (chromene C7), 154.6 (chromene C8α), 161.2 (C=O at chromene C3), 161.7 (chromene C2), 171.2 (NHC=O), 171.7 (OC=O). ESI-MS m/z 361 [M+^1^]. Anal. Calc. for C_18_H_20_N_2_O_6_ (%): C 59.99; H 5.59; N 7.77. Found (%) C 59.78; H 5.49; N 7.71.

*(S)-Ethyl 5-(ethylamino) -5-oxo -2-(2-oxo-2H-chromene-3-carboxamido) pentanoate* (3b). White solid, m.p. = 68−70 °C, Yield: 66.8%. ^1^H-NMR (CDCl_3_): 1.11 (t, 3H, *J* = 7.2 Hz, CH_3_CH_2_NH), 1.28 (t, 3H, *J* = 7.1 Hz, CH_3_CH_2_O), 2.17–2.28 (m, 3H, COCH_2_CH_2_CHCO), 2.31–2.40 (m, 1H, COCH_2_CH_2_CHCO), 3.23–3.28 (m, 2H, CH_3_CH_2_NH), 4.10–4.13 (m, 1H, COCH_2_CH_2_CHCO), 4.20 (q, 2H, *J* = 7.1 Hz, CH_3_CH_2_O), 5.61 (br s, 1H, NH), 6.90 (dt, 1H, *J* = 7.5, 0.8 Hz, chromene H6), 6.97 (d, 1H, *J* = 8.2 Hz, chromene H8), 7.28 (dd, 1H, *J* = 7.7, 1.6 Hz, chromene H5), 7.34 (dt, 1H, *J* = 8.5, 1.6 Hz, chromene H7), 8.39 (s, 1H, chromene H4), 12.96 (br s, 1H, NH).^13^C NMR: 14.2 (CH_3_CH_2_NH), 14.8 (CH_3_CH_2_O), 29.1 (COCH_2_CH_2_CHCO), 32.1 (COCH_2_), 34.4 (CH_2_NH), 61.5 (OCH_2_), 70.2 (CHCO), 117.7 (chromene C8), 118.5 (chromene C3), 118.9 (chromene C4α), 131.9 (chromene C4, C6), 132.9 (chromene C5, C7), 161.0 (chromene C8α), 167.4 (chromene C2, C=O at chromene C3), 171.0 (NHC=O), 171.4 (OC=O). ESI-MS m/z 375 [M+^1^]. Anal. Calc. for C_19_H_22_N_2_O_6_ (%): C 60.95; H 5.92; N 7.48. Found (%) C 60.77; H 5.69; N 7.39.

*(S)-Ethyl 5-(ethylamino)-5-oxo-2-(6-fluoro-2-oxo-2H-chromene-3-carboxamido)pentanoate* (3c). White solid, m.p. = 65−67 °C, Yield: 71.5%. ^1^H-NMR (CDCl_3_): 1.10 (t, 3H, *J* = 7.2 Hz, CH_3_CH_2_NH), 1.28 (t, 3H, *J* = 7.1 Hz, CH_3_CH_2_O), 2.16–2.25 (m, 3H, COCH_2_CH_2_CHCO), 2.29–2.40 (m, 1H, COCH_2_CH_2_CHCO), 3.18–3.28 (m, 2H, CH_3_CH_2_NH), 4.12–4.15 (m, 1H, COCH_2_CH_2_CHCO), 4.20 (q, 2H, *J* = 7.1 Hz, CH_3_CH_2_O), 5.96 (br s, 1H, NH), 6.90 (dd, 1H, *J* = 9.0 Hz, *J*_HF_ = 4.4 Hz, chromene H8), 6.99 (dd, 1H, *J*_HF_ = 8.3 Hz, *J* = 3.0 Hz chromene H5), 7.05 (dt, 1H, *J*_HF_ = 10 Hz, *J* = 3.8 Hz, chromene H7), 8.34 (s, 1H, chromene H4), 12.74 (br s, 1H, NH).^13^C NMR: 14.0 (CH_3_CH_2_NH), 14.6 (CH_3_CH_2_O), 29.0 (COCH_2_CH_2_CHCO), 31.8 (COCH_2_), 34.2 (CH_2_NH), 61.4 (OCH_2_), 70.0 (CHCO), 116.7 (*J*_CF_ = 22.9 Hz, chromene C5), 117.9 (chromene C4α), 118.0 (chromene C4), 118.1 (chromene C3), 119.8 (*J*_CF_ = 23.2 Hz, chromene C7), 155.0 (*J*_CF_ = 235.4 Hz, chromene C6), 156.9 (C=O at chromene C3), 166.27 (chromene C8α), 166.29 (chromene C2), 170.7 (NHC=O), 171.3 (OC=O). ESI-HRMS m/z 347.1406 [M-EtO]. Anal. Calc. for C_19_H_21_FN_2_O_6_ (%): C 58.16; H 5.39; N 7.14. Found (%) C 58.01; H 5.22; N 6.95.

*(S)-Ethyl 5-(ethylamino)-5-oxo-2-(6-nitro-2-oxo-2H-chromene-3-carboxamido)pentanoate* (3d).

Light yellow solid, m.p. = 165−167 °C, Yield: 84.6%. ^1^H-NMR (CDCl_3_): 1.14 (t, 3H, *J* = 7.2 Hz, CH_3_CH_2_NH), 1.31 (t, 3H, *J* = 7.1 Hz, CH_3_CH_2_O), 2.13–2.21 (m, 1H, COCH_2_CH_2_CHCO), 2.30–2.45 (m, 3H, COCH_2_CH_2_CHCO), 3.25–3.32 (m, 2H, CH_3_CH_2_NH), 4.24 (q, 2H, *J* = 7.1 Hz, CH_3_CH_2_O), 4.76–4.81 (m, 1H, COCH_2_CH_2_CHCO), 6.32 (br s, 1H, NH), 7.60 (d, 1H, *J* = 9.0 Hz, chromene H8), 8.54 (dd, 1H, *J* = 9.0, 2.5 Hz, chromene H7), 8.73 (d, 1H, *J* = 2.5 Hz, chromene H5), 9.04 (s, 1H, chromene H4), 9.18 (br d, 1H, *J* = 7.7 Hz, NH).^13^C NMR: 13.9 (CH_3_CH_2_NH), 14.5 (CH_3_CH_2_O), 28.2 (COCH_2_CH_2_CHCO), 32.2 (COCH_2_), 34.2 (CH_2_NH), 52.4 (OCH_2_), 61.7 (CHCO), 117.8 (chromene C8), 118.3 (chromene C3), 119.9 (chromene C4α), 125.6 (chromene C4), 128.3 (chromene C6), 144.3 (chromene C5), 147.4 (chromene C7), 157.4 (chromene C8α), 159.5 (C=O at chromene C3), 160.3 (chromene C2), 170.9 (NHC=O), 171.1 (OC=O). ESI-MS m/z 420 [M+H], 442 [M+Na], 861 [2M+Na]. Anal. Calc. for C_19_H_21_N_3_O_8_ (%): C 54.41; H 5.05; N 10.02. Found (%) C 54.19; H 5.00; N 9.87.

### Detection of *in vitro* and *in vivo* fluorescent signals

We injected TFC, TNC (80 mg/kg, i.p.) or DMSO as a control into C57/BL6 mice. Three hours later, the fluorescent imaging *in vivo* and *in vitro* (TFC or TNC at 8 mg/ml in a tube) was recorded under 530 nanometer excitation and 600 nanometer emission. We obtained images on a Kodak Image Station 4000 Multi-Modal Imaging System (IS4000MM) equipped with an X-ray unit and on a Kodak Image Station 2000 (Carestream Health, Rochester, NY, USA.).

### Preparation of sera from TFC- and TNC-fed rats

These were done according to our published methods with slight modifications [10; 19] Briefly, SD rats (age range, 6 week; from Tumor Research Institute, Chinese Academy of Sciences, Beijing, China) were treated in accordance with guidelines established by the Animal Care and Use Committee at Yantai University (ACUC) and were approved by the ACUC at Yantai University, China. TFC or TNC were orally administered to the rats once daily at a dose of 80 mg/ml/kg body weight. The blood was then collected at 0, 1, 3, 5, and 8 h from the rats (fasted for 16 h) after oral intubation of TFC or TNC. The collected blood was left to clot for 2 h at room temperature and centrifuged twice at 3000x g at 4ºC for 20 minutes. The sera were sterilized by filtration and then heated at 56 ºC for 30 minutes. The prepared sera were allocated, and stored at -80°C until *ex vivo* growth assay.

### Cell culture and *in vitro* & *ex vivo* growth assay

The cell lines of human lung cancer A549 and NCI-H460 (H460), human leukemia K562, highly metastatic Lewis lung cancer (LLC), and normal human embryonic lung fibroblast (MRC-5) were obtained from the American Type Culture Collection. Human peripheral blood lymphocytes (PBL) were separated as the reported method [27]. The CSCs were from Shandong DEBO Biotechnology Polytron Technologies Inc. (Jina, Shandong, China). The cell lines of A549, H460, LLC, MRC-5, and K562 as well as PBL were cultured in DMEM and RPMI 1640 medium (for K562 and PBL only), respectively, containing 10% heat-inactivated fetal bovine serum (FBS), glutamine (2 mM), penicillin (100 U/ml) and streptomycin (100 μg/ml) at 37ºC in a humidified incubator with 95% air/5% CO_2_ atmosphere. The rate of cell growth was determined using MTT assay or Trypan blue exclusion test (TBET) according to our published methods [19-20; 23]. Cells were seeded in 96-well plates (Becton Dickinson, NJ, U.S.A.) at 2x10^3^ per well (1x10^5^ per well in the case of PBL) and incubated overnight to allow attachment. The cells in control group were treated with DMSO [0.1% (v/v), final concentration]. The cells were incubated in DMEM or RPMI 1640 medium supplemented with 10% FBS containing different concentrations of TMC, TEC, TFC or TNC (0.016 to 0.25 mM), or Ly294002 (Ly, 0.016 mM), Bay (0.0032 mM), or in combination of existing anticancer drugs cytarabine (10-25 nM), vincristine (50-100 μM), and methotrexate (10-25 nM), or 10% rat sera (in the case of *ex vivo* assay) obtained at different time points after TFC and TNC was orally intubated the rats. Ly and Bay are the inhibitors of PI3K/Akt, and NF-*κ*B, respectively. After 48 and 72 h of treatment, the absorbance values in each test group were measured using MTT assay. For the test of cancer stem cells (CSCs), the CSCs were isolated from human lung A549 cells and identified by FACS for the protein expressions of positive CD133 and other cancer stem cell surface markers as well as the spheres-forming capacity and other CSCs characteristics. The CSCs at 0.6x10^4^ per well at 96-well were treated with TFC and TNC at the concentrations of 16 to 250 μM. After 4-day treatment, the absorbance values in each test group were measured using MTT assay. The relative cell growth was calculated based on the absorbance of sample vs. the absorbance of control (vehicle). Experiments were performed in triplicate. In the case of TNFα- or EGF plus HGF-treated cells, A549 cells at 3x10^5^ per well at 6-well were treated with TFC and TNC for 30 minutes. Then the cells were treated with TNF-α(20 ng/ml) and EGF (100 ng/ml) plus HGF (100 ng/ml) for 24 h, respectively. The collected cells were analyzed for the viability and protein expressions by TBET and Western Blotting, respectively. Each experiment was performed in triplicate and repeated at least three times.

### Flow cytometry for cell apoptosis

The effects of TFC and TNC on cell apoptosis were analyzed according to the instructions of Annexin V-FITC/PI Apoptosis Detection Kit [28]. In brief, cells were treated for 24 h with TFC or TNC (0.25 mM), 3-methyladenine (3M, 12.5 μg), or Z, Z-VAD-FMK (12.5 μM), or in combination of TFC or TNC with 3M or Z. The cells in control group were treated with DMSO [0.05% (v/v), final concentration]. The treated cells were stained with Annexin V-FITC and propidium iodide (PI). The apoptosis was analyzed by flow cytometry (Becton Dickinson FACS Vantage SE, San Jose, CA, USA). Twenty-thousand events were analyzed per sample and the total apoptosis ratio including the early and late apoptosis were determined.

### Migration assay

Tumor cell migration was measured by examining that the amount of cells pass through a fibronectin-coated polycarbonate filter, using modified transwell chambers as described previously [19-20; 29]. In brief, cells (5 x 10^4^) were seeded onto the upper chamber in 200 μL of serum-free medium containing different concentrations of TMC, TEC, TFC or TNC (0.004 to 0.016 mM); the lower compartment was filled with 0.66 mL of DMEM media supplemented with 10% of FBS (as a chemoattractant). The cells in control group were treated with DMSO (0.1%, final concentration). After incubation for 6 h at 37°C, the cells that migrated to the lower surface of the filter were fixed and stained using propidium iodide. The cells on the upper side of the filter were removed using a rubber scraper. The migrated cells on the underside of the filter were counted and recorded for images under a fluorescent microscope (Nikon, TE2000-U, Japan). Experiments were performed in triplicate.

### Western blot analysis

Cells were treated with different concentrations of TFC or TNC (0.016 to 0.25 mM), Ly294002 (Ly, 0.016 mM), or Bay (0.0032 mM). The cells in control group were treated with DMSO [0.1% (v/v), final concentration]. The treated cells were collected either at 30 minutes for detection of phosphorylation ratios of pEGFR/EGFR, and pAkt/Akt, or at 48 hours for detection of the protein expression of Bcl-2, Bax, procaspase/caspase-3, PARP-1, cyclin D1, p21WAF1, p53, NF-κB (p65), VEGFR1, VEGFR2, and cytosolic cytochrome c using our published methods with some modifications [19-20; 30]. Whole cellular proteins were extracted, and the cytosolic fraction proteins were prepared following the procedure described by the manufacturer (Beyotime, Haimen, China). The protein concentration of the extracts was determined using the Bradford method. Equal amounts of cell extracts were resolved by SDS-PAGE, transferred to nitrocellulose membranes, and probed with the primary antibodies to the detected proteins mentioned above, and then horseradish peroxidase-conjugated secondary antibodies, respectively. Anti-β-actin antibody was used as a loading control. Detection was done using an enhanced chemiluminescence system (GE Healthcare Life Sciences, Piscataway, NJ USA).

### Cell fractionation and electrophoretic mobility shift assay (EMSA)

A549 cells were treated for 30 minutes with TFC and TNC at 0.25 mM. After 6 h of TNF-α treatment, the treated cell were then pelleted by centrifugation at 1,000 rpm for 5 min at 4°C and resuspended in ice-cold buffer A (10 mM HEPES (pH 7.9), 10 mM KCl, 0.1 mM EDTA, 1 mM DTT, 0.5 mM phenylmethysulfonylfluoride (PMSF), 1 μg/mL leupeptin, 5 μg/mL aprotinin). Following the addition of 25 μL 10% NP40, the suspension was vortexed and centrifuged at 14,500 rpm for 1 min at 4°C; the supernatant was designated as the cytoplasmic fraction. Nuclei were resuspended in 50 μL of ice-cold buffer B (20 mM HEPES (pH 7.9), 0.4 M NaCl, 1 mM EDTA, 1 mM DTT, 1 mM PMSF, 25% glycerol, 1 μg/mL leupeptin, 5 μg/mL aprotinin) and centrifuged at 14,500 rpm for 5 min. The supernatant was used as the nuclear fraction and protein concentration determined by the Bradford method. Electrophoretic mobility shift assay (EMSA) was done with 10-μg nuclear protein using the Biotin 3′ End DNA Labeling Kit (Pierce, Rockford, Product # 89818) and Biotinlabeled NF-κB consensus oligonucleotide (5'-AGT TGA GGG GAC TTT CCC AGG C-3'). Nuclear protein-DNA complexes were separated by 4% PAGE. Transferred DNAs were UV cross-linked to the membrane and detected using horseradish peroxidase-conjugated streptavidin (LightShift^TM^ chemiluminescent EMSA kit, Pierce, Rockford, Product # 20148) according to the manufacturer's instructions.

### Subcutaneous tumor model

Female BALB/c *nu/nu* mice and C57/BL6 mice (age range, 6 weeks) were purchased from Tumor Research Institute, Chinese Academy of Sciences, Beijing, China). All animal procedures were carried out in accordance with the guidelines established by the Animal Care and Use Committee (ACUC) at Yantai University and were approved by the ACUC at Yantai University, China. Single-cell suspensions containing 3 × 10^6^ A549 or 0.6 × 10^6^ LLC cells in 0.1 mL Hank's balanced salt solution were injected s.c. into rear right flank of each BALB/c *nu/nu* mouse (in the case of A549 cells) or each C57/BL6 mouse (in the case of LLC cells), respectively. After 10-15 days, the tumor models were established. The tumor-bearing mice were randomly divided into four groups (six and seven mice pre group for LLC and A549 experiments, respectively): (1) control group (vehicle); (2) cisplatin group for LLC tumor treatment (1.5 mg/kg/day; 6 days each week); endostar group for A549 tumor treatment (7 mg/kg/day; 6 days each week); (3) TFC group (TFC 80 mg/kg/day); (4)TNC group (TNC 80 mg/kg/day). The mice were treated with vehicle, cisplatin, endosatr, TFC and TNC by introperitoneal injection once every day. The therapy was performed for 15 days (in the case of LLC tumors) and 28 days (in the case of A549 tumors), respectively. Tumor volumes were measured every two days (LLC tumors) or three days (A549 tumors). On the 15th or 28th day, mice in all groups were sacrificed and tumors were weighed. Results were plotted as relative tumor weight and growth (volume) for the first day of the treatment up to the final day [31]. The relative tumor weight is relative to that of the control (vehicle) designated as 100.

### Statistical analysis

The data were expressed as mean ± standard deviation (S.D.) and analyzed by the SPSS 16.0 software to evaluate the statistical difference. Statistical analysis was done using the ANOVA and Bonferroni post-test. Values between different treatment groups at different times were compared. The rate (%) of relative cell migration, growth, and mean protein levels as well as the relative tumor volumes and weights are shown for each group. For all tests, *P* values less than 0.05 were considered statistically significant. All statistical tests were two-sided.

### List of Abbreviations used

TMC, methyl coumarin-3-carboxylyl L-theanine; TEC, ethyl coumarin-3-carboxylyl L-theanine; TFC, ethyl 6-fluorocoumarin-3- carboxylyl L-theanine; TNC, ethyl 6-nitrocoumarin-3-carboxylyl L-theanine; NF-*κ*B, nuclear factor *κ*B; VEGFR, vascular endothelial growth factor receptor; EGF, epidermal growth factor; EGFR, epidermal growth factor receptor; HGF, hepatocyte growth factor; Ly, Ly294002, Bay, Bay 11-7082; PARP, poly(ADP-ribose) polymerase; 3M, 3-methyladenine; Z, Z-VAD-FMK; TBET, Trypan blue exclusion test; CSCs, cancer stem cells; MTT, 3-[4,5-Dimethylthiazol-2-yl]- 2,5-diphenyltetrazolium bromide; Cyto C, cytochrome c; LLC, Lewis lung cancer; EMSA, electrophoretic mobility shift assay.
